# Phenomenographic Approaches in Research About Nursing

**DOI:** 10.1177/23333936231212281

**Published:** 2023-11-25

**Authors:** Martha M. Whitfield, Mike Mimirinis, Danielle Macdonald, Tracy Klein, Rosemary Wilson

**Affiliations:** 1 Queen’s University, Kingston, ON, Canada; 2 University of Vermont, Burlington, VT, USA; 3 University of Liverpool, Liverpool, United Kingdom; 4 University of West London, London, Middlesex, United Kingdom; 5 Washington State University Vancouver, Vancouver, WA, USA

**Keywords:** phenomenography, qualitative methodologies, nursing research, variation theory, qualitative interviews, conceptions

## Abstract

We propose that phenomenography is well-suited to research about nursing, given its focus on identifying variation in individuals’ experiences, and inclusion of diverse voices and perspectives. Phenomenography explores qualitatively different ways in which a group of people experience a phenomenon, often using semi-structured interviews. The use of phenomenography is especially relevant in research about nursing which provides accounts of the experiences of nurses and patients within complex practice settings. We consider the tenets of phenomenography and examine phenomenography’s relationship to and differences from phenomenology. We review literature published about phenomenographic research in nursing and reflect on the potential benefits of phenomenographic research about nursing. This paper adds to knowledge about use of phenomenography in research about nursing.

Phenomenography has been proposed as a viable qualitative methodological approach for exploring a wide range of issues in healthcare settings and as an appropriate choice for building healthcare knowledge, including within nursing (Barnard et al., 1999; Röing & Sanner, 2015; Röing et al., 2018). While healthcare researchers have used phenomenography in a variety of settings, there is relatively little recent writing about the implications of choosing to conduct phenomenographic research within nursing.

Sjöström and Dahlgren (2002) provided a helpful overview of the application of phenomenography in nursing, which they proposed is especially suited to developing understanding about nursing, given the potential focus on variation in patient experiences as well as student conceptions. Phenomenography has also been proposed as an approach for research about clinical decision making (Baker, 1997) and nursing education (Barry et al., 2017; McClenny, 2020). Twenty years after publication of Sjöström and Dalgren’s paper, nurses’ work occurs in increasingly complex and rapidly changing environments. The continued growth of advanced practice nursing globally sees nurses working in roles with extended scopes of practice, requiring higher order decision-making skills. Understanding nurse and patient experiences continues to be important for nursing as a discipline, and for optimizing patient care. Phenomenographic studies, and those in which researchers use a “phenomenographic approach” are proliferating. A recent search of Medline and CINAHL for “phenomenography” and “nursing” yielded almost 200 published studies since 2002. However, relatively few authors have written about the use of phenomenography as a methodological approach to research about nursing or by nurses. Aside from Sjöström and Dahlgren (2002), we identified an overview of phenomenography for nursing researchers (Jobin & Turale, 2019), literature reviews focused on research in nursing education and learning by nursing students (Barry et al., 2017; McClenny, 2020), and a critique of phenomenographic approaches to research in nursing (Friberg et al., 2000).

Here we reflect on the use of phenomenographic approaches in research done by nurses and about nursing. We begin by outlining the foundations of phenomenography, and briefly discuss its relationship to phenomenology. We then describe the basic tenets of phenomenographic studies and discuss the relevance of phenomenographic research in healthcare with a focus on nursing. We discuss ways in which rigor and trustworthiness are conceived and accounted for in phenomenographic research, as well as the implications for these criteria in research about nursing.

While phenomenography was initially used in educational settings, and much discussion of phenomenography has occurred in the context of education (Cibangu & Hepworth, 2016; Marton & Booth, 1997), phenomenography has a history of broad application, including in healthcare (Barnard et al., 1999). Since many researchers in nursing and healthcare are choosing a phenomenographic approach, it is important to continue to discuss phenomenography as a choice of methodology within these settings. This paper contributes to knowledge about the use of phenomenography in research by nurses and about nursing and articulates the potential of phenomenography as a research approach for authors looking at clinical decision-making, student nursing and learning, patient experiences, and advanced practice nursing.

## Background

### Premises of phenomenography

Phenomenography’s development as a qualitative research approach originated from the work of Ference Marton and other educational researchers working at the University of Gothenburg in the late 1970s (Barnard et al., 1999). Phenomenography has been described as a research approach rather than a research method (Marton & Booth, 1997; Tight, 2015), although the literature refers variously to phenomenography as a methodology, method, and approach. For example, McClenny (2020) uses the terms method and methodology interchangeably in an overview of phenomenography in nursing education research.

Phenomenographic research focuses on the qualitatively different ways of experiencing a phenomenon. Researchers explore people’s relationship or experience with phenomena (Marton, 1986), examining variation in how participants think about and conceive their experiences (Marton & Booth, 1997; Sjöström & Dahlgren, 2002). Conceptions are expressed in a set of categories of description (Barnard et al., 1999; Trigwell, 2006). A focus on the collective, rather than the individual experience is used to identify variation in how a phenomenon is experienced by, and within, a group (Beaulieu, 2017; Sjöström & Dahlgren, 2002; Trigwell, 2006), and iterative analysis is used to reveal qualitative differences within a group of individuals experiencing the same phenomenon (Beaulieu, 2017; Trigwell, 2006). The focus of phenomenography on variation or differences within the collective experience of a group of participants distinguishes it from other approaches.

Phenomenography assumes a second-order, relational perspective, describing people’s relationship with phenomena, rather than observing or describing a phenomenon directly (Marton, 1986). Ontologically, phenomenography proposes a non-dualistic approach which assumes there is no division between the internal world we experience as individuals and the external world (Marton, 1981; Marton & Booth, 1997; Sjöström & Dahlgren, 2002; Trigwell, 2006). The unit of phenomenographic research comprises an examination of the internal relationship between what is experienced (the phenomenon) and the person(s) who are experiencing it. This internal relationship is a central tenet of phenomenography as described by Marton and Booth (1997) where reality is neither “constructed by the learner, nor. . .imposed upon her; it is *constituted* as an internal relation between them” (p.13). Researchers do not seek the essence of an external reality or truth; rather participants live within the world that they experience, without a separation between internal and external realities (Marton & Booth, 1997). While objects exist outside of experience, it is assumed that it is only possible for them to be described in terms of what is experienced (Marton & Booth, 1997). Thus, a research program should seek to discern the ways in which people think about and interpret the world by understanding their experiences (Marton, 1981). Since individuals experience reality differently, phenomenographic research is aimed at examining variation in experiences (Linder & Marshall, 2003; Sjöström & Dahlgren, 2002), including how variation in “aspects” of a phenomenon contributes to its definition (Marton & Booth, 1997).

In a phenomenographic study, categories of description depict the qualitatively different ways in which a phenomenon is understood; the logical and structural relationship between these categories constitutes the *outcome space* of the phenomenon (Marton, 1986; Mimirinis et al., 2023). Within the outcome space the more advanced categories at the top of the hierarchy include elements of those at lower levels, but not the reverse. Thus, researchers consider not only variation in understanding, but also the structure within which variation can be understood (Åkerlind, 2023b).

### Developments in phenomenography—toward variation theory

Phenomenography has developed as a methodological approach since its inception in 1980s, and researchers continue to explore new avenues (Rovio-Johansson & Ingerman, 2016). Variation theory represents a shift in the focus of inquiry from qualitative differences in understanding of a phenomenon to explorations of what makes learning possible (Wright & Osman, 2018). In variation theory, phenomena are considered in contrast to or variation with others, or against an invariant background (Åkerlind, 2023b; Marton, 2015). Learning occurs in the context of the ability to discern difference (Marton, 2015; Rovio-Johansson & Ingerman, 2016). Phenomenography and variation theory are closely entwined but have distinct applications; together, their value has been proposed as pedagogical framework with potential for transformation in higher education (Wright & Osman, 2018). Learning studies apply variation theory within learning environments in a cyclical format, to study and refine teaching practice (Rovio-Johansson & Ingerman, 2016). An explanation of the differences between phenomenography, variation theory, and learning studies was offered by Rovio-Johansson and Ingerman (2016):

Phenomenography explores the qualitatively different ways in which people potentially ‘experience’ certain phenomena they meet in their worlds, variation theory offers a framework for understanding what it takes to experience something in a certain way (or learn about it), and learning studies make use of that framework to design teaching for good learning results. (p. 261)

In phenomenography, the focus is on variation within the group experience of a constant phenomenon; in variation theory the focus is on the “object of learning,” or what should be learned, and the ways in which this is perceived or understood. The “critical aspects” of an object of learning are those aspects which a learner must be able to discern to master the object of learning (Rovio-Johansson & Ingerman, 2016). The object of learning itself is not always static, but rather may have dynamic properties in the context of teaching and learning (Rovio-Johansson & Ingerman, 2016). Variation may apply to the object of learning as a whole, and to individual critical aspects. In phenomenography critical aspects can be used to define elements of the outcome space to indicate qualitatively different perceptions of a phenomenon; in variation theory critical aspects are the dimensions of variation which can be focused upon by learners; while in learning studies, critical aspects are seen as essential to the learner’s ability to fully discern the object of learning (Pang & Ki, 2016).

### Relationship to and differences from phenomenology

As qualitative methodologies, phenomenography and phenomenology share several features. Marton (1981) acknowledged phenomenography’s debt to the long history of phenomenology and pointed to phenomenology for the historical development of phenomenography, describing relational, experiential, contextual, and qualitative features that are shared between the two approaches (Marton, 1986, p. 40). However, while the origins of phenomenography can be traced to phenomenology, and some see phenomenography as a phenomenological “subset” (Cibangu & Hepworth, 2016), Marton (1986) clarified that he did not conceive of phenomenography as derived directly from phenomenology, but rather as a pragmatic approach to inquiry about teaching and learning. Thus, phenomenography is separate from phenomenology (Barnard et al., 1999).

For phenomenographic researchers, phenomena are generally experienced in a relatively limited number of ways (Marton, 1981, p. 181). The “essence” of a phenomenon is central to phenomenology; in phenomenography, researchers are not concerned with determining the essence of a concept or phenomenon. While phenomenologists are concerned with commonalities, or with those aspects that define a phenomenon, phenomenographers seek to consider the variation in how individuals conceive or experience their relationship with a phenomenon (Marton, 1986; Trigwell, 2006). Thus, a phenomenographic exploration of nursing could encompass variation in any aspect of how nursing is conceptualized or experienced. Of course, such a broad remit for phenomenography means that variation may extend to the practice of phenomenography itself (Åkerlind, 2012).

An interesting approach to debating phenomenology versus phenomenography was taken in a study where both phenomenographic and phenomenological analyses were applied to one set of interview data focused on how anesthesiologists conceive their work (J. Larsson & Holmström, 2009). J. Larsson and Holmström (2009) used each methodological approach in turn to analyze their interview data, implying that the data itself could serve both analytical approaches. However, differences between phenomenology and phenomenography became apparent in both the focus of the research question and the results of the analysis (J. Larsson & Holmström, 2009). Taking a phenomenographic approach, rather than asking “what is anesthesiology?,” the researchers asked “what do experienced anesthesiologists think about what anesthesiology is?” (J. Larsson & Holmström, 2009, p. 57). The results of the J. Larsson and Holmström (2009) study illustrated this shift in focus: the phenomenographic analysis resulted in four categories, labeled metaphorically, and focused on varying aspects of role perception, while the phenomenological analysis described the anesthesiology role and profession itself.

### Phenomenography in research about nursing and by nurses

A key challenge in healthcare is the requirement to respond to diverse patient needs (Sjöström & Dahlgren, 2002). For example, because patients and clinicians can understand and interpret medical diagnoses, treatments, and care plans in different ways, a phenomenographic approach can be helpful in teasing out and better understanding these differences and their clinical implications (Stenfors-Hayes et al., 2013). It is important for nurses and other clinicians to take such variations between patients into account in their clinical work (Sjöström & Dahlgren, 2002). Phenomenographic study of patient experiences may help us discern the critical aspects of learning about chronic conditions such as diabetes, where a patient’s understanding of their illness and approach to self-care may significantly impact outcomes. Authors of previous phenomenographic studies have explored healthcare research generally (Barnard et al., 1999); the patient experience (Frank et al., 2009; Röing & Sanner, 2015); medical education (Fyrenius et al., 2007; Stenfors-Hayes et al., 2013); and issues in nursing (Sjöström & Dahlgren, 2002).

Understanding the thinking a nurse applies as they conceptualize patient care is an important step in determining how clinical decision-making is learned and applied. The ability to gain insight into the nurse’s conceptions of patient care with a focus on the development of how nurses evolve as clinical decision makers has been proposed to be a good fit for phenomenography over other qualitative methods in research about nursing (Baker, 1997). A more complete understanding of patient conceptions can help clinicians think about the patient experience and how best to provide information and education to patients (Sjöström & Dahlgren, 2002; Stenfors-Hayes et al., 2013).

Phenomenography’s roots in the field of education make it an appropriate methodological approach for exploring the perspectives of nurse educators or for considering how novice nursing students or health professionals move toward more expert conceptual understanding (Han & Ellis, 2019; Sjöström & Dahlgren, 2002). Phenomenography has also been proposed as helpful in uncovering variations in student nurses’ understanding of their learning, and in evaluating the effectiveness of teaching methods when addressing challenging concepts in nursing (Barry et al., 2017). Research about how nurses conceptualize their clinical decision-making skills may reveal how such skills are developed and assist clinical educators with teaching strategies (Baker, 1997; Sjöström & Dahlgren, 2002; Stenfors-Hayes et al., 2013). For example, Fyrenius et al. (2007) explored how understanding is achieved in higher education generally and considered aspects of learning and understanding specific to medical contexts and problem-based learning.

Research about patient learning and education is also important for nursing. While we may make assumptions about what patients learn when given education about their health, patient learning can depend on several factors (Frank et al., 2009; I. Larsson et al., 2019). Just as a phenomenographic researcher attempts to understand what students are doing in their learning (Trigwell, 2006), nurses may find it useful to understand what patients or clients are experiencing or taking away from their learning. For example, Frank et al. (2009) used open-ended interviews, so that patients could identify which aspects of how they experienced participation in their own care they wanted to discuss. Given that the patient experience may not correlate with the experience of the nurse or other healthcare provider, an openness of questioning seems key—in other words patients should be able to talk about what *they* find important—which may not be what is initially deemed most important from a nursing or medical perspective.

Phenomenographic researchers have explored the conceptions and experiences of nurses with advanced education and roles, including doctoral students (Arvidsson & Franke, 2013), nursing researchers and academics (Dupin et al., 2015; Forbes, 2011; Letterstål et al., 2022), and clinical supervisors or nurse managers (Dyar et al., 2021; Hyrkäs et al., 2003). However, we located few phenomenographic studies published in English where the participants were advanced practice nurses (APNs) or had an advanced scope. Some researchers considered the experiences of registered nurse anesthetists (Knudsen et al., 2022; Mauleon & Ekman, 2002; Nordström & Wihlborg, 2019; A. Tracy, 2017), while authors of one study addressed the nurse practitioner experience (Lin et al., 2021). Understanding and describing conceptions about what constitutes advanced practice is important given continued momentum toward adoption of APN roles in a range of settings and contexts globally (World Health Organization, 2020). Many APNs practice autonomously, and most jurisdictions provide for their ability to prescribe medication. Phenomenography therefore has potential as an approach for exploring and articulating the diversity and complexity of APN practice.

## Data Collection

Semi-structured interviews are the most used method of data collection in phenomenographic research, although other data collection methods can be used (Baker, 1997; Bruce, 1994). While Bruce (1994) proposed that certain features distinguish a phenomenographic interview from other qualitative interviewing techniques, phenomenographic interviews are similar in many respects to those conducted in other qualitative methodologies, including phenomenology (Barnard et al., 1999). The participant’s conceptions may include what makes up the phenomenon and how various aspects of the phenomenon are related, sometimes called the internal horizon; and a broader understanding of how the phenomenon exists and is discerned as being within but separate from its surrounding context, sometimes called the external horizon (Linder & Marshall, 2003, p. 273; Marton & Booth, 1997). The phenomenographic interviewer’s intent is to determine a participant’s internal and external horizon regarding the structure of the phenomenon being explored, while maintaining a focus on seeking variation in the ways in which a phenomenon is conceptualized.

Consistent with a second-order perspective, the interview focus is on the participant’s relational experience of a phenomenon and interview questions focus on how the phenomenon is experienced, understood, or perceived (Bruce, 1994). Interview questions in phenomenography are generative, and can focus on “why”—for example “why do you say that, or why is it so”—and “what”—for example “what does that mean to you?” (Säljö, 1979). Sjöström and Dahlgren (2002) proposed that the interview guide comprise a few opening questions with the remainder of the interview devoted to follow-up of the answers elicited. For example, Andersson et al. (2015) began all interviews with the question “Please tell me what caring means to you in your clinical work as a nurse?’’ (p.3), while Lin et al. (2021) asked nurse practitioners “What was work like today?” (p. 211).

Booth (1997) described interviews as both “open” and “deep”—meaning that the discussion may diverge from any predetermined plan to include new avenues, and the conversation is continued until both participant and the interviewer feel that it has been fully explored. The interviewer can encourage participants to reflect on their answers by pausing to consider and potentially question their responses (Sjöström & Dahlgren, 2002). Interview questions are aimed at revealing the participant experience of the phenomenon by providing as much freedom as possible for participants to share and reflect deeply on their experiences, meanings, and understandings (Pang & Ki, 2016). However, as in other qualitative interviews, the researcher should keep the phenomenon under investigation central to their thinking, demonstrate curiosity, and use probing questions to circle back to the central question or phenomenon during the interview as needed. The interview can be conceived as a central question around which the conversation orbits. If the conversational orbit grows larger or wobbles, the interviewer’s role is to gently nudge it back as close to the central question as possible. Asking participants to think back to a clinical scenario may allow them to reflect on their actions, for example by asking participants to think back to an early encounter with a patient, to describe it, and to reflect on their learning and things that might have changed.

The “open” and “deep” phenomenographic interview process (Booth, 1997) has parallels to the clinical history-taking interview, especially as conducted by an advanced practice nurse or physician. In a clinical interview, a nurse or other healthcare provider may focus on getting a patient history relevant to the central question at hand (abdominal pain for example) but must be open to unanticipated answers that reveal new clinical information. However, within the time limits of a patient visit in the clinic setting, the clinical interviewer must necessarily learn to make quick decisions about what aspects are most relevant or require further exploration. Unlike the phenomenographic interviewer, the interviewer in a clinical setting may not have the luxury of pursuing the discussion to its fullest extent. In addition, phenomenographic researchers aim to set aside their own theories and to focus on how interview participants understand a phenomenon. This differs from the clinical interview, in which the nurse not only listens attentively to the patient’s experience but must simultaneously be engaged in considering the potential cause of what the patient is reporting.

The interviewer working within nursing or healthcare may require some content expertise, including being cognizant of relevant vocabulary (Stenfors-Hayes et al., 2013). For example, an interviewer may need to be conversant with current regulations, pharmaceutical formularies, medical and nursing guidelines, and specialist vocabulary. It may be necessary to adapt interview techniques depending on the interview participants. Researchers may need to vary the language used, depending on whether participants are patients, healthcare providers, family members, or support staff, with consideration of literacy and education levels.

## Data Analysis

As part of the phenomenographic analysis, researchers complete iterative readings of the collated interview transcripts. *Meaningful utterances*, that is those parts of the transcripts that tell the researcher something about the structure of the conceptions of the phenomenon under investigation, are selected and together make up a *pool of meanings* (Marton, 1986). Based on similarities and differences, the researcher groups and re-groups these meaningful utterances until they form a set of categories of understanding about the phenomenon. Tentative categories of description are re-compared to the pool of meaningful utterances, and categories adjusted until a limited set of logically, internally, and hierarchically related categories of description have been defined (Mimirinis, 2019; Mimirinis & Ahlberg, 2021). These categories should stand in “clear relation to the phenomenon” and each should tell us something unique or “distinct” about one way of experiencing the phenomenon (Marton & Booth, 1997, p. 125).

The process of data analysis is iterative as the researcher(s) move(s) back and forth between preliminary categories and the pool of meanings, adjusting as needed. As noted, categories of description are hierarchically structured, and are inclusive; thus, categories higher in the hierarchy incorporate elements of preceding categories. Conceptions within the categories may have both referential aspects, which consider meaning and are relational to the larger context; and structural aspects, which concern the elements and structure of the conception. Dimensions of variation may be present across the categories of description but can change within each category (Mimirinis, 2019).

A higher level of understanding within the categories of description may be correlated with increasing expertise or capability in practice. For example, Knudsen et al. (2022) found three ways of understanding airway management algorithms amongst registered nurse anesthetists, where the third category was correlated with practice at an expert level, and the ability to apply algorithms flexibly, taking the clinical scenario into account. In a meta-synthesis of phenomenographic studies looking at the understanding of work by health professionals, Röing et al. (2018) identified five qualitatively different categories of understanding. The understanding of work ranged from a focus on the individual healthcare provider and the challenges of providing care to patients, to a more nuanced and holistic understanding of patient care within the larger healthcare system (Röing et al., 2018). In nursing, the patient–nurse relationship is both central and essential. By adopting a second-order perspective researchers can explore the qualitatively different experiences of patients, with implications for nurses and others who assume professional roles in healthcare. For example, patient perceptions of self-determination within the context of care were explored by Nordgren and Fridlund (2001), who found that patients perceived nurses as having potential to support them in more active involvement in their own care.

## Ensuring Rigor and Trustworthiness

Ensuring quality and rigor from the outset in phenomenographic research starts with determining the fit of phenomenography for a research question and continues to the final analysis (Sim, 2010). Given nursing’s focus on applicability, nursing researchers may find it helpful to refer to the components of quality in qualitative research outlined by S. J. Tracy (2010): worthy topic, rich rigor, sincerity, credibility, resonance, significant contribution, ethics, and meaningful coherence. S. E. Thorne (2016) discussed ensuring trustworthiness through attention to disciplinary relevance, pragmatic obligation, contextual awareness, and probable truth. Purposive sampling is used for phenomenographic studies to try to ensure as much variation among participants as is feasible (Mimirinis, 2022). However, it can be difficult to accurately determine differences between participants prior to the interviews (Stenfors-Hayes et al., 2013). In healthcare research generally, additional ethical considerations may exist, for example, when interview participants are also patients, when sensitive health information is being discussed, or when participants may not be able to give full informed consent (Coleman, 2019). Moreover, only participants who experienced a phenomenon can be selected and meaningfully engaged with the process of a phenomenographic interview.

The trustworthiness of a phenomenographic interview can be established through the clarity and explicitness of the interview questions (Sjöström & Dahlgren, 2002). Asking participants to reflect on what they mean by interview statements can help clarify their intentions (Sim, 2010). If the interviewer holds a similar nursing or clinical role to interview participants, participants may assume understanding by the researcher. However, it may be necessary to ask probing or clarifying questions even in this instance. The interviewer may ask for details or information that they might be assumed to know in other settings.

Being a clinical “insider” as well as a researcher can legitimize the researcher for participants, with assumed collegiality contributing positively to the interview. However, it is also critical to maintain an awareness of the researcher role, to ensure that an interview does not devolve into a conversation between peers, and to avoid making assumptions about meaning. While Säljö (1997) describes data collection as collecting information about what people say, it is important to distinguish the interview from conversation so as not to interject personal responses as a researcher. Yet, as in most qualitative interviews, the interviewer does steer the conversation, not only by asking questions, but by choosing what points to ask clarifying questions about, or where to probe more deeply.

*Bracketing*, or striving to set aside one’s assumptions about the research data to maintain as much objectivity as possible, can be problematic in research about nursing which assumes that nurses practice according to an ethical code (S. Thorne et al., 2016). Bracketing of the researcher’s own experience and conceptions is not a focus for those working in phenomenography (Marton, 1986; Sim, 2010). Rather than bracketing their responses to the data, the researcher focuses on the empirical data—their analysis is concerned with the findings in the data, rather than with interpretation. Thus, a phenomenon cannot be described as separate from the person experiencing or describing it (Marton & Booth, 1997). In phenomenography the researcher is not looking to interpret the data, but to find similarities and differences within it, to uncover critical aspects of the experience of a phenomenon as it is described by the interview participants. Therefore, the tendency to editorialize or interpret must be resisted. However, as nurse researchers, one cannot be fully separated from the data. It can be difficult to separate the experience of a phenomenon as described by a participant, from the experience of being asked to describe this as part of an interview (Dortins, 2002). Researchers must maintain an “interpretive awareness,” adopting a critical approach to considering their own subjectivities and interpretations (Åkerlind, 2012, 2023a).

Interpretation or application follows the empirical findings, meaning that phenomenography could be suited to asking just the type of questions that S. Thorne et al. (2016) proposed, for example considering how knowledge about diversity of experience can provide insight to nurses as they interact with patients. Similar questions have been proposed as ideally answered by phenomenography—where researchers seek to understand the ways in which individuals experience the real-life situations in which they find themselves, and problems they are grappling with (Marton & Booth, 1997). Like interpretive description (S. E. Thorne, 2016; S. Thorne et al., 2016), phenomenography is also proposed as an approach to tackling questions about teaching and learning that are situated within a specific context (Marton & Booth, 1997).

Having just one researcher complete the interviews has been proposed as key to maximizing consistency in how the data itself is obtained for phenomenography (Green & Bowden, 2009). However, reliability may be enhanced by having more than one researcher involved in the analysis of the transcripts so that idiosyncratic interpretations of the data are avoided (Trigwell, 2006). Researchers can analyze the data independently and compare results with another researcher or can come to consensus through discussion (dialogic reliability) (Åkerlind, 2012). Categories of description should be replicable given the same data set but would be expected to vary with different data or a different set of interviews. As with all qualitative methodologies the researcher should be able to defend their choices for categories based on the data. Pilot interviews can be used to test whether the interview questions elicit meaningful and relevant responses and to review interview techniques (Andersson et al., 2015).

Even while attempting to maintain impartiality, what the interviewer does and does not say will shape the interview, including what elements of the interviewee’s description they pick up on and how they ask participants further clarify or expand on their responses. Dortins (2002) describes this process as a “negotiation” and the interviews as “collaborative endeavors” (p.209).

In the case of a conceptual topic, where participants may have spent years thinking about and reflecting on their work, interviews can generate a large amount of data. Sorting through the data requires decisions about what to include or exclude, and as researchers we may play a role in shaping what is presented. For example, Dortins (2002, p. 208) talked about editing herself “out of” the interviews as she transcribed them. Trying not to read anything into the transcripts that is not stated can be challenging, as one reads more deeply and repetitively. Despite our best efforts to keep personal interpretation out of the interview and analysis process, as interviewers we are bound to influence participant responses and their analysis.

## Ethical Considerations

Kvale and Brinkman (2009) addressed some of the ethical issues in interviewing in the health sciences in their seminal work on qualitative interviewing—largely in reference to the influences of the movement for evidence based practice and in discussion of the use of ethical review boards as a practice that grew out of biomedical research; however, they do not offer guidelines for researchers conducting interviews with individuals who work in healthcare, or discuss phenomenography specifically. Interviews with patients, or where participants discuss interactions with patients may entail the sharing of sensitive information. Therefore, transcripts should be de-identified, including removing any references that might situate a participant in a particular clinic, as well as any patient identifiers. Nurses are generally well-aware of the need for confidentiality related to patient information, and comfortable with sharing de-identified cases as part of their participation in case reviews, communities of practice, or in discussion with mentors. Nonetheless, patient and nurse confidentiality must always be preserved. Ethical considerations may also arise in instances where the researcher and participants have an established relationship, especially when a power differential is present. For this reason, Mauleon and Ekman (2002) made the choice to use open-ended written questions rather than interviews with newly graduated nurse anesthetists, in recognition of inherent power imbalances in the student–teacher relationship.

## Discussion

### Considering the Use of Phenomenography in Research About Nursing

Research about nursing encompasses a broad range of topics and foci, reflecting the breadth of nursing as a discipline. Several factors influence qualitative nursing research design, including nursing knowledge (both general and particular); the dynamism and complexity of current healthcare environments; and nursing’s “moral mandate and action imperative” (S. Thorne et al., 2016, p. 451). S. Thorne et al. (2016) proposed that a “nursing disciplinary epistemology”—yet still evolving—be used in designing qualitative research about nursing (p. 455), and further than nursing itself contains the elements required to create a “credible frame” for applied qualitative research (S. Thorne et al., 2016, p. 458). Moulton et al. (2019) argued that a central question for nursing would provide a rationale and validation for research about nursing, and proposed that this central question might be “how can the well-being of a person, family, community, or population be improved?” (p.1). To understand how nursing and nurses might answer this question, the experience of both nurse and patient must be considered within a larger environmental context.

What defines nursing itself may differ depending on the nursing context, the patient, the level of nursing education, and authorization granted to the nurse in policy and law. Nursing assumes a relationship between the nurse and the patient, where the patient may be an individual, family, or community. The nurse–patient relationship is often structured around a particular focus of care, with a resulting a triad of nurse, patient, and care. While Moulton et al. (2019) acknowledged that well-being and improvement have multiple potential interpretations, they suggested that this is not an issue in proposing a central question for nursing; it is up to nursing researchers to clarify the specifics of their inquiry.

It is important that nursing research knowledge be applicable to the practice of nursing (S. Thorne et al., 2016). S. Thorne et al. (2016) identified areas of disconnect or disciplinary tension between nursing knowledge and traditional qualitative methodologies, as well as outlining the historical reluctance on the part of nursing researchers to adopt applied methodologies that might open them up to criticism about the rigor and trustworthiness of their work (S. Thorne et al., 2016). As a situated practice, nursing bridges both the natural and human sciences, since nursing knowledge is “developed for practice” (Moulton et al., 2019, p. 4) and is used for the benefit of improving the health of individuals or groups (S. Thorne et al., 2016). In considering phenomenography as a useful methodology for examining nursing, we must consider what methodologies should be used to develop nursing knowledge (Moulton et al., 2019) and how those methodologies should be used within applied disciplines such as nursing (S. Thorne et al., 2016).

Commenting on the challenges of applying qualitative methodologies to nursing, S. Thorne et al. (2016) noted that while phenomenology can provide researchers with a way of examining facets of how healthcare and illness are experienced, its use may also result in a potential lack of appreciation of the “intricate variation” and “human diversity” experienced by nurses in the course of their work (p. 453). Like phenomenology, phenomenography shines a research focus on the subjective experiences of health. However, phenomenography also allows researchers to place variation in experiences among nurses and patients at the center of their explorations (Sjöström & Dahlgren, 2002). There is no inherent judgment about the quality of analysis or findings, rather the nursing researcher must consider what questions they want to ask, and to what end when making a methodological choice.

In critically assessing phenomenography, some have pointed out that researchers can only categorize variation in the *descriptions* of experience since they lack access to the experience itself (Richardson, 1999; Säljö, 1997). Although phenomenography is generally understood to be concerned with experience, Säljö (1997) proposed that phenomenography explores the discourses related to what people say rather than what they experience, an observation that could also be applied to other methodological approaches. When looking for meaningful utterances however, we may also ask ourselves whether it is possible to separate the language and expression of the interview subject from that of the interviewer? Säljö (1997) noted that meaningful utterances are considered as indicative of ways of experiencing but questioned whether in fact these utterances might be better understood as ways of talking about a phenomenon, or even ways of responding to a question out of a sense of obligation. Thus, qualitatively different ways of experiencing may in fact be qualitatively different ways of talking about a phenomenon (Säljö, 1997). Säljö (1997) described phenomenography as one approach (among many) to describe thinking in a way that does not separate an external reality from a person’s internal thoughts about that external reality, by adopting a non-dualist approach. Some have also expressed concerns about difficulties reconciling phenomenographic conceptions within their broader contexts in nursing and other “caring research” given the inherent complexity of this area of enquiry (Friberg et al., 2000).

## Implications and Conclusions

We propose that a thoughtful and pragmatic application of existing methodologies, including phenomenography, can allow nursing researchers to benefit from the richness of existing research approaches. With its empirical focus on identifying variation, phenomenography can help nursing researchers explore how both nursing practice and care is conceptualized. Phenomenographic researchers intentionally encourage the inclusion of diverse voices and worldviews, often elicited through in-depth interviews. The incorporation of diverse perspectives can help to ensure that those whose experience might otherwise be marginalized are included. Additionally, phenomenography’s use as a broad methodological approach allows for its application to diverse research questions, including exploration of both teaching and learning in nursing (Barry et al., 2017).

Understanding the variation in how participants experience a phenomenon is especially relevant in nursing, where nurses, patients, and other healthcare staff interact within settings that are inherently complex and often unpredictable. Phenomenography can be a useful approach to the discipline of nursing broadly, and in developing knowledge that can be applied to practice whether from the perspective of practice, education, or research, and encompassing a broad range of nursing roles including that of clinician, student, teacher, educator, or leader. A more complete understanding of patient conceptions can help clinicians think about the patient experience and how best to provide information and education to patients. Likewise, clinician conceptions of key issues in nursing are important in understanding the experiences of nurses within the workforce. A phenomenographic approach can also reveal gaps in understanding, which may be helpful to policy makers and educators, including those concerned with workforce retention and development. Thus, the focus on variation in experience that underpins phenomenography makes it an especially apt methodology for the exploration of how nursing practice accommodates and incorporates variation in clinical scenarios and the practice environment.
